# Influence of SARS-CoV-2 pandemic on sleep habits in a pediatric population

**DOI:** 10.5935/1984-0063.20210021

**Published:** 2022

**Authors:** Sara Completo, Andreia Fiúza Ribeiro, Ana Rute Manuel, Helena Cristina Loureiro

**Affiliations:** Hospital Professor Doutor Fernando Fonseca, E.P.E., Pediatric Service, Child and Youth Department - Amadora - Amadora - Portugal

**Keywords:** COVID-19 Pandemic, SARS-CoV-2, Sleep Habits, Electronic Devices, Lockdown

## Abstract

**Objective:**

Assessment of changes in sleep habits at home in children during COVID-19 lockdown.

**Methods:**

Retrospective, transversal study in a pediatric ward of a level II hospital. Questionnaires concerning sleep quality, patterns and its modifications during lockdown were distributed from June to August 2020. Comparison with a control sample from previous study (2019). Statistical analysis on SPSS Statistics23.

**Results:**

Two groups were compared: during lockdown (n=36, mean age 9.3 years-old) and before lockdown (n=48, mean age 8.8 years-old). 55.6% stated changes in sleep patterns. There was an increase in sleep hours, specifically in school-aged children (p=0.05) and adolescents (p=0.03), with no impact in global subjective sleep quality. Significative increase in screen hours (p=0.02) and its use after dinner (p=0.04).

**Discussion:**

Changes in sleep patterns during lockdown were frequent, alongside a higher use of screens. However, these did not affect the subjective sleep quality nor increased the occurrence of sleep disturbances.

## INTRODUCTION

Coronavirus disease 2019 (COVID-19) pandemic affected children and adults worldwide, not only for the disease burden but also for the mandatory confinement it demanded, which disrupted daily routines and activities. Children are shaped by the environment where they grow, which influences long-term mental and physical health, thus being seemingly more susceptible to the constraints required by the pandemic.

During school closure, children and adolescents experienced a significant decline in all physical activities, mainly outdoor activities and sport, accompanied by increased screen time and social media usage^1^. Though the shift to online and remote learning can be accountable for a rise in electronic devices use, evidence shows that its contribution is not as significant as leisure activities to the sedentary behavior observed in children and adolescents, a trend exacerbated during the pandemic^2^. These changes seem to be more pronounced in girls and adolescents, placing them at greater risk of obesity, diabetes, and cardiovascular disease, as these new behavioral habits may become permanently rooted^2^.

COVID-19 related restrictions are also likely to impact sleep. For once, less physical activity and changes in sleep schedules and pre-sleep routines are known factors associated with poorer sleep^3^. Moreover, shortening of sunlight exposure alongside increased exposition to artificial lights and blue lights from screens can destabilize the circadian cycle with a negative effect on sleep^4^. Also, health concerns, changes in family financial situations, and uncertainty about the future may subject children and adolescents to higher stress and anxiety levels^5^.

Since good quality sleep is paramount for a functional immune system and overall health, it is reasonable to assume that variations in sleep patterns during the COVID-19 pandemic may be related to a higher susceptibility to SARS-CoV-2 infection and severe disease^6^.

This study aimed to assess the change in sleep habits and sleep quality at home of children and adolescents during the COVID-19 pandemic and compare them with the sleep habits before the pandemic, which results come from a previous study performed in the pediatric ward during the year of 2019.

## MATERIAL AND METHODS

This was a retrospective and transversal study developed in a pediatric ward of a public level II hospital in Amadora, which serves 155,628 pediatric patients, according to the Portuguese census of 2011, with a hospitalization rate of around 1.0% in 2019.

A questionnaire, comprising closed and open questions, was distributed to in-patients in the pediatric ward (when age was above or equal 12 years old) or their caregivers (when in-patient age was between one and 12 years old), over six weeks (between the end of June and early August of 2020). The questionnaire was based on another one from a previous study, with additional questions about the habits during the lockdown period. The mandatory confinement in Portugal lasted from 18^th^ March to 2^nd^ May 2020, and during this time schools were closed and there were scarce permissions to leave the house.

The questionnaire assessed family setting, whether family members worked from home, were in lay-off from work or unemployed, and whether children or adolescents were in a homeschooling regime. It evaluated sleep habits at home, its modifications since lockdown and the global subjective sleep quality, measured by two variables: the average nighttime awakenings and the subjective sleep quality set by each participant on a quantitative scale from 0 (very bad) to 10 (very good). The use of screens during the period of confinement was also assessed.

Participants were randomly selected in the pediatric ward among in-patients above one-year-old. Children with cognitive disorders that impaired the sleep-wake cycle or whose caregivers did not consent to the study were excluded.

A previous study was conducted on the pediatric ward of the same hospital in 2019 to compare the sleep conditions at home and during hospitalization. The home sleep conditions were collected through the same questionnaire and the results were used as a control sample to compare the habits before and during lockdown.

Statistical analysis was performed with SPSS® v.23.0 (SPSS Inc, Chicago, IL, U.S.), using parametric and non-parametric tests (independent t-test, chi-square test) and the level of significance *p*<0.05, to compare the two groups: before and during COVID-19 lockdown.

The project was approved by the ethical committee of our institution. Informed consent was given to all participants. All information was anonymous and confidential.

## RESULTS

### Sleep at home during lockdown

This study included 36 participants, of whom 52.8% were male, with a mean age was 9.3 years old. Eighty percent attended daycare/school previous to the beginning of the SARS-CoV-2 pandemic.

Considering sleep evaluation, 55.6% identified changes in sleep patterns during lockdown, mostly characterized by later bedtime and waking time schedules, not only during the week but also during the weekend. Of these, 20.0% reported restless sleep and 30.0% admitted having changed the usual bedtime routine. Ten percent had increased diurnal somnolence. It was observed statistically significant changes in sleep patterns in females (*p*<0.01). Only 11.1% reported more difficulty falling asleep and 5.6% had more nightmares related to the pandemic.

The mean sleep hours per day for each age group, including naptime, is shown in Table 1 and fulfils the American Academy of Sleep recommendations for each case^7^. The children and adolescents enrolled in the study reported, on average, 0.8±1.1 nighttime awakenings and a mean subjective sleep quality of 8.5±1.3 on a scale from 0-10.

**Table 1:** Comparison of the sociodemographic characteristics, sleep conditions and screens usage between the two samples (before and during lockdown).

Demographic characteristics	During lockdown (n=36)	Before lockdown (n=48)	p
Average age	9.3	8.8	0.66
	(min. 18 mon; max. 17 y)	(min. 12 mon; max. 18 y)	
Age group			0.13
Preschoolers	30.6%	33.3%	
School-aged children	16.7%	33.3%	
Adolescents	52.8%	33.3%	
Gender			0.75
Male	52.8%	56.3%	
Female	47.2%	43.7%	
Maternal literacy			0.71
Primary school	0.0%	4.2%	
Basic education	36.1%	35.4%	
High school	30.6%	29.2%	
Bachelor’s degree	22.2%	18.8%	
Master’s degree	8.3%	4.2%	
At least one parent unemployed	19.4%	12.5%	0.46
At least one parent working from home	22.2%	N/A	
At least one parent in lay-off from work	16.7%	N/A	
**Sleep conditions at home**			
Sleeps in individual room	38.9%	37.5%	0.90
Has company when falling asleep	47.2%	45.8%	0.96
Falls asleep in their own bed	83.3%	87.5%	0.34
Naptime among children under 6 y	81.8%	100.0%	0.08
Average duration (hours)	1.9h	1.5h	0.10
Average nighttime awakenings	0.8 ± 1.1	0.9 ± 1.0	0.91
Average subjective sleep quality (scale 0-10)	8.5 ± 1.3	8.9 ± 1.3	0.22
**Screens usage**			
After dinner	88.9%	68.8%	**0.04**
Online/remote school	58.4%	N/A	
At least one sibling in online/ remote school	66.6%	N/A	
**NA: not applicable**			

The majority of participants (83.3%) engaged in more activities with screens since the beginning of lockdown, with several purposes: 38.9% for online/remote school, 36.1% to watch movies and videos, 30.6% to chat with friends, and 13.9% for social networking. Particularly, it was observed a significant increase in screen usage between adolescents (*p*=0.02). Most participants (47.2%) spent four hours or more per day using screens and 88.9% used electronic devices after dinnertime.

Table 1 summarizes the answers obtained from the questionnaires regarding demographic characteristics, sleep conditions at home, and screens usage.

### Sleep at home before lockdown

These results are part of a previous study used here as a control group. It included 48 participants, of whom 56.3% were male, with a mean age was 8.8 years old.

The mean sleep hours per day for each age group, including naptime, is shown in Table 1. It was reported, on average, 0.9±1.0 nighttime awakenings and a mean subjective sleep quality of 8.9±1.3 on a scale from 0-10.

Per day 20.8% spent four hours or more using screens and in 68.8% electronic devices were used after dinner time.

### Comparison between the two groups

**Figure 1:**
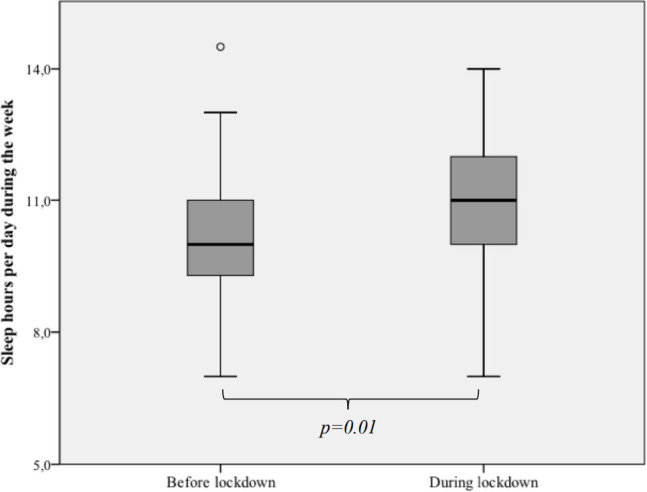
Comparison of the median hours of sleep per day, during the week, before and during lockdown.

**Figure 2:**
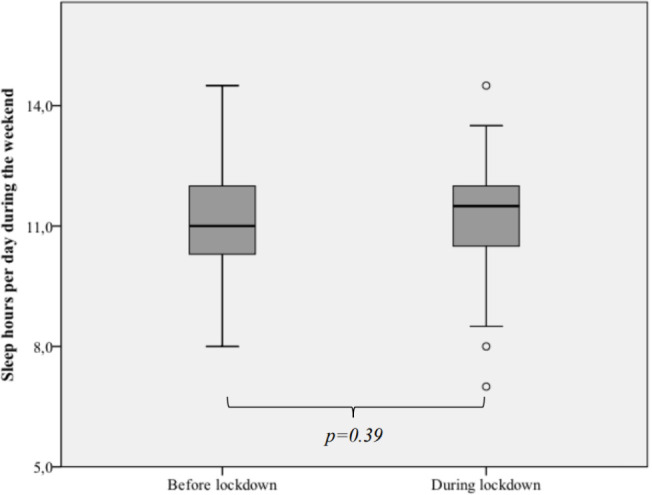
Comparison of the median hours of sleep per day, during the weekend, before and during lockdown.

The two samples were compared and had similar sociodemographic characteristics (Table 1). During the COVID-19 pandemic, there was an increase in sleep hours during the week, specifically in the school-aged children (on average 53.8 minutes, *p*=0.05) and adolescents (on average 71.5 minutes, *p*=0.03), but no significant change in the sleep hours during the weekend and naptime (Figures 1 and 2). Also, there was a substantial decrease in the sleep hours gap between the week and weekend (from 56.4 minutes before lockdown to 16.8 minutes during lockdown, *p*=0.03).

There was no difference in global subjective sleep quality during the pandemic, since there was no change in the average nighttime awakenings (*p*=0.91) nor in the mean subjective sleep quality (*p*=0.22).

**Figure 3:**
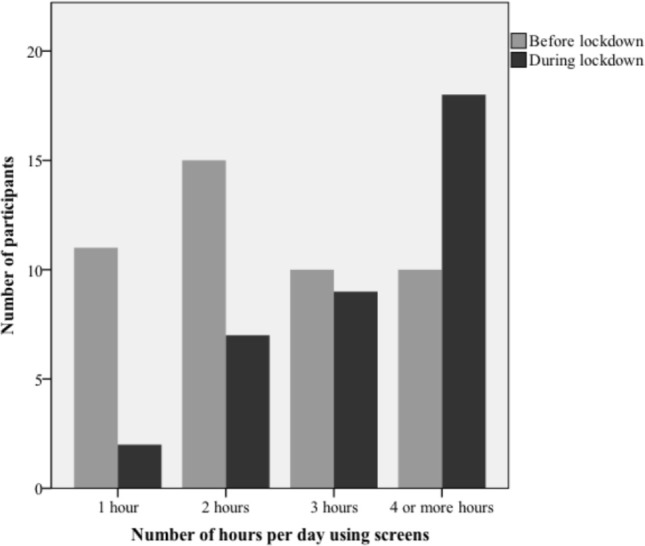
Number of hours per day spent using screens, before and during lockdown.

Additionally, there was a significant increase in the number of screen hours (*p*=0.02 - Figure 3) and the use of screens after dinner (*p*=0.04) during lockdown.

## DISCUSSION

This article adds preliminary results concerning the sleep of children and adolescents during the lockdown required by the COVID-19 pandemic. Until this moment, few studies have been published on this topic in the pediatric population and results remain controversial about the effect of lockdown on sleep quality, though generally agreeing on a modification of sleep patterns^8-15^.

Appropriate timing, duration, quality, regularity, and absence of sleep disturbances are all necessary characteristics for a healthy sleep^7^.

Our results showed a significant percentage of participants identifying changes in their sleep patterns, mainly characterized by later bedtime and waking time schedules, a finding common to other papers^12-14^. These changes were more significant in the female group. In addition, it was observed that school-aged children and adolescents had a significant increase in sleep duration throughout the week, a difference that was not significant during the weekend. This may be justified by the fact that adolescents already slept more hours during the weekend before the pandemic, which also explains the reduction in the sleep hours gap between the week and weekend in lockdown. The increase in sleep length during the week was seen to a lesser extent in preschoolers and was not statistically significant. Moreover, it was observed that less children under the age of six had naptime, though still achieving the recommended sleep hours^7^. This may be explained by the lack of schedules and routines in the absence of kindergarten/school.

Despite the variations observed in sleep timing and duration in lockdown period, there was no impact on the subjective sleep quality expressed by participants. One possible explanation is that individuals benefit from the opportunity of a more appropriate sleep pattern, with personally regulated hours, reducing social jetlag, and respecting their own circadian rhythm, with no impairment of sleep quality^5,6^. Findings in literature diverge on this subject^8,10,11^. Of note, Zreik et al. (2020)^11^ demonstrated an association between poorer subjective sleep quality in children of mothers with greater acute COVID-19 anxiety and insomnia symptoms^11^. This emphasizes the role of parental support and environmental factors in sleep quality, which may, in part, be responsible for the differences seen across studies^16^. Besides, the effect of the pandemic on sleep quality can also differ according to age, as found by Zhou et al. (2020)^10^ that described a great likelihood of sleep quality decline and sleep disturbances in college students compared to junior and senior school students.

Regarding sleep disturbances, no difference in the nighttime awakenings was found and only a small percentage reported difficulty falling asleep, restless sleep, and diurnal somnolence. Actually, a prospective international study found that although lockdown can produce anxiety-induced insomnia and other sleep disorders, this situation is rare and usually short-term^15^. Furthermore, evidence shows that lesser knowledge about COVID-19 relates to a higher rate of insomnia symptoms among adolescents^10^.

Our findings showed an important increase in electronic device usage, particularly in adolescents, with the majority of the participants using screens after dinnertime, a practice that is commonly discouraged^17^. One can inquire if this greater consumption of screens may be related to the delay and the changes in bedtime routine observed in a percentage of cases.

This study has some limitations. In one hand, it has a limited number of participants, justified by the reduced hospitalization rate during COVID-19 pandemic. In the other hand, the study sample is not the same as the control sample. However, this does not appear significant since no differences were found in the sociodemographic data and the sleep-conditions at home between the two samples.

In conclusion, this study showed that changes in sleep patterns during lockdown were frequent, alongside a higher use of electronic devices. However, this did not seem to affect the subjective sleep quality of children and adolescents, and did not increase the occurrence of sleep disturbances significantly.

Further investigation should focus on the long-term analysis of the role of the COVID-19 pandemic on pediatric sleep, especially on sedentarism and screen consumption and their influence on sleep patterns. Moreover, it would be important to evaluate how the lack of schedules and routines, alongside anxiety and insomnia related to COVID-19, can impact the subjective sleep quality in the near future, and also its long-term implications on the health of children and adolescents.

### Previous presentations

This study was presented as a poster on the “*1^as^ Jornadas Digitais de Pediatria*”, in October 2020, in Portugal.
